# Cardiac progenitors derived from reprogrammed mesenchymal stem cells contribute to angiomyogenic repair of the infarcted heart

**DOI:** 10.1007/s00395-012-0301-5

**Published:** 2012-10-18

**Authors:** Stephanie Buccini, Khawaja Husnain Haider, Rafeeq P. H. Ahmed, Shujia Jiang, Muhammad Ashraf

**Affiliations:** Department of Pathology and Laboratory Medicine, University of Cincinnati, 231 Albert Sabin Way, Cincinnati, OH 45267-0529 USA

**Keywords:** Bone marrow, Heart, iPS cells, Mesenchymal, Pluripotency, Reprogramming

## Abstract

The strategy to reprogram somatic stem cells to pluripotency status has provided an alternative source of surrogate ES cells (ESC). We report efficient reprogramming of multipotent bone marrow (BM) mesenchymal stem cells (MSC) to pluripotent status and the resultant MSC derived iPS cells (MiPS) and their derived progenitors effectively repaired the infarcted heart. MSC from young, male, Oct4-GFP transgenic mice were reprogrammed by retroviral transduction with Oct4, Sox2, Klf4, and c-Myc stemness factors. MiPS thus generated displayed characteristics of mouse ESC including morphology, surface antigens, gene and miR expression profiles. MiPS also formed spontaneously beating cardiac progenitors which expressed cardiac specific transcription factors and protein markers including Gata4, Mef2c, Nkx2.5, myosin heavy chain, troponin-I, and troponin-T, and showed ultra structural characteristics typical of cardiomyocytes. Intramyocardial delivery of MiPS (group-2) and their derivative cardiac-like cells (MiPS-CP; group-3) in a mouse model of acute myocardial infarction showed extensive survival and engraftment at 4 weeks with resultant attenuation of infarct size (*p* < 0.001 vs. DMEM injected control; *n* = 4). Engraftment of MiPS-CP was without cardiac tumorigenesis as compared to 21 % in MiPS transplanted animals. Furthermore, angiogenesis was improved in groups-2 and 3 (*p* < 0.001 vs. control). Transthoracic echocardiography revealed significantly preserved indices of cardiac contractility (ejection fraction *p* < 0.001 and fractional shortening *p* < 0.001 vs. control; *n* = 7). MSC were successfully reprogrammed into MiPS that displayed ESC-like characteristics and differentiated into spontaneously beating cardiomyocytes. Cardiac progenitors derived from MiPS repopulated the infarcted heart without tumorigenesis and improved global cardiac function.

**Electronic supplementary material:**

The online version of this article (doi:10.1007/s00395-012-0301-5) contains supplementary material, which is available to authorized users.

## Introduction

The cytokine rich microenvironment of the infarcted myocardium infiltrated by inflammatory cells and activation of various signaling pathways as part of the intrinsic repair mechanism remains a major challenge in cardiovascular therapeutics [22, 27, 38]. In this regard, heart cell therapy involving mobilization of stem cells from an intrinsic pool in the body or by transplantation of stem cells as an outside intervention has shown significant promise as a therapeutic modality for ischemic heart disease [6, 7, 11, 15, 29, 30]. Given the multilineage differentiation capacity and autologous availability, bone marrow (BM) derived mesenchymal stem cells (MSC), with or without genetic modification, have been extensively studied for myocardial reparability [7, 26, 29]. Besides, paracrine release of bioactive molecules by MSC contributes to improved heart function via pro-survival effects on the host cardiomyocytes and initiation of angiogenic response in the infarcted myocardium [36]. We hypothesized that reprogramming of MSC would improve their cardiomyogenic capabilities and proposed that induced pluripotent stem cells (iPSC) derived from MSC (MiPS) would be more amenable to differentiation into cardiac progenitors (CP). Furthermore, MiPS would be superior to fibroblast-derived iPSC (FiPS) as they might carry forward the epigenetic memory of myogenic and angiogenic traits.

We report successful reprogramming of MSC using the classical four-factor cocktail into MiPS which were identical to mouse ESC and displayed potential for cardiac differentiation in vitro by forming spontaneously beating clusters of cardiac progenitors. Similar to previous reports, we observed that MiPS engrafted efficiently to prevent left ventricular (LV) remodeling and preserved global heart function [2, 20]. However, unlike Nelson et al., we observed formation of teratomas in the heart after undifferentiated MiPS transplantation in immunocompetent mice. This is consistent with our previous findings using skeletal myoblast derived iPSC [1]. Therefore, differentiation of MiPS into cardiac progenitors (MiPS-CP) curtailed their tumorigenicity and significantly enhanced their engraftment capacity which led to reduced LV remodeling, attenuated infarct size and improved heart function. Similar to native MSC, MiPS-CP also displayed an improved angiogenic response in the infarcted heart. We found no teratomas in MiPS-CP transplanted animal hearts due to altered expression of cardiac differentiation-related and tumor-suppressive genes and miRs, respectively, thus demonstrating that differentiation of MiPS into CP may be safer and more applicable for stem cell therapy.

## Materials and methods

The present study conformed to the guide for the care and use of laboratory animals published by the US National Institutes of Health (NIH Publication No. 85–23, revised-1985) and protocol approved by the Institutional Animal Care and Use Committee, University of Cincinnati.

### Isolation and characterization of MSC

MSC were purified from young (4–6 weeks) male, Oct4-GFP transgenic mice (Jackson Laboratories, USA) with GFP tagged to endogenous Oct4 gene promoter as detailed in Supplementary data. Briefly, bones were isolated and flushed with high glucose Dulbecco’s modified Eagle’s medium (DMEM; 10 % fetal bovine serum and 0.5 % penicillin/streptomycin) [36]. The adherent MSC were propagated and maintained in high glucose DMEM at 37 °C, 5 %CO_2_ atmosphere. The cells were maintained and expanded for no more than three passages before use in subsequent experiments.

The adherent cells were harvested with 0.025 % trypsin (Sigma Aldrich, MO, USA) and analyzed by flow cytometry (BD FACSCanto, BD Biosciences, NJ, USA) for surface marker expression as detailed in Supplementary data.

### Generation of iPSC

Retroviral vectors encoding for the Yamanaka stemness factors Oct4, Sox2, Klf4 and C-Myc were purchased from Addgene Inc., USA (plasmid #13367, #13366, #13370, #17220 from Takahashi et al.) [34]. The viruses were used for transduction of mouse embryo fibroblasts (MEF: Global Stem, MD, USA) and MSC for generation of FiPS and MiPS, respectively. Plasmids for each of the pluripotency factors and the envelope protein VSV-G plasmid were transduced into GP2-293 packaging cells using CalPhos Mammalian Transfection Kit (Clontech, CA, USA). After 72 h, supernatant was collected from each of the four vectors, mixed together and centrifuged at 1,200 rpm for 5 min. The combined viral supernatant was passed through a 0.45 μm cellulose acetate filter and used for transduction of MSC or MEF. Briefly, 24 h before retroviral infection, low passage MSC or MEF (passage 2–3) were seeded in 6-well plates at a density of 2 × 10^5^ cells/well. The cells were incubated for 42 h in supernatants containing the vectors encoding for the stemness factors followed by culture for 48 h in fresh complete growth medium. The cells were re-plated in a 10 cm culture dish with irradiated MEF on 0.1 % gelatin using mouse ESC medium. Mouse ESC medium was replenished every other day as cells were observed for growth of iPSC clones [2]. After 18–21 days, iPSC clones displaying ESC-like morphology were mechanically isolated and cultured on irradiated MEF. The iPSC clones were propagated individually in ESC culture medium.

Clones of iPSC were cultured on irradiated MEF (5 × 10^4^ cells/cm^2^) in ESC culture medium that consisted of Knockout™ DMEM supplemented with 15 % Knockout™ serum replacement, 0.5 % penicillin/streptomycin, 0.1 mmol/L non-essential amino acids, 0.2 mmol/L l-glutamine, 0.1 mmol/L mercaptoethanol (Invitrogen, CA, USA), and 10^3^ U ml^−1^ leukemia inhibitory factor (LIF; Millipore, CA, USA). The iPSC were characterized by alkaline phosphatase staining, immunocytochemistry [1], DNA methyltransferase activity assay, teratoma formation and karyotyping as described in Supplementary data.

### In vitro cardiac differentiation of MiPS

MiPS (2 × 10^6^ cells/10 ml medium) were grown in suspension for 3 days in high glucose DMEM containing 10 % FBS, 0.5 % penicillin/streptomycin, 0.1 mmol/L non-essential amino acids, and 0.1 mmol/L mercaptoethanol) [2]. After 3 days in suspension, embryoid bodies (EBs) were plated on 0.1 % gelatin-coated tissue culture dishes (500–1,000 EBs/10 cm dish) and cultured for another 7–10 days. Spontaneously contracting regions were mechanically isolated and dissociated for cardiac specific markers, ultra-structure studies and miR expression profiling as described in Supplementary data.

### In vitro studies of anoxia-induced angiogenic factors

Native MSC, MiPS, MiPS-CP, FiPS or MEF were serum-starved for overnight. Cells were treated with two 30 min cycles of anoxia with one 10 min re-oxygenation in between. Control cells for each group were maintained at normal oxygen levels. The cells were immediately harvested and analyzed by RT-PCR for fibroblast growth factor (FGF) and vascular endothelial growth factor (VEGF) expression.

### Experimental animal model of coronary artery ligation and MiPS transplantation

An acute myocardial infarction model was developed in 8–12 week-old immunocompetent female C57BL/6 mice [36]. Animals were anesthetized (Ketamine/Xylazine 0.05 ml intraperitoneally) and intubated. The heart was exposed by minimal left sided thoracotomy and coronary artery was permanently ligated. The animals were grouped to receive intramyocardial injections of 20 μl basal DMEM without cells (control), or 2 × 10^5^ MSC (group-1), MiPS (group-2), MiPS-CP (group-3), FiPS (group-4), or MEF (group-5). The cells were labeled with red fluorescent Q-tracker^®^-625 (Invitrogen, CA, USA) to study the fate of the cells post-transplantation in the infarcted heart. Heart function was determined by transthoracic echocardiography (Supplementary data). The animals were harvested for histological studies at stipulated time-points.

### Histological studies

Histological studies were performed as previously described [2]. To measure infarction size, the hearts were arrested in diastole by intravenously injecting them with cadmium chloride followed by fixation in 10 % formalin. The hearts were cut transversely and embedded in paraffin. Histological sections were cut at 6 μm thickness and were stained with Masson’s trichrome staining to visualize fibrosis. Cryosections were fixed with 4 % paraformaldehyde in PBS for 10 min and later incubated with CAS-BLOCK (Invitrogen, CA, USA) for 1 h at room temperature. Slides were then incubated with primary antibody overnight at 4 °C (Supplementary Table-II). Slides were then washed in PBS and primary antigen–antibody reaction was detected using goat anti-rabbit Alexa Fluor-488 conjugated secondary antibody and goat anti-mouse Alexa Fluor-488 conjugated secondary antibody (1:200, Invitrogen, CA, USA).

### Statistical analysis

All data presented were described as mean ± SEM. One-way analysis of variance with Holm-Sidak method was used to determine significant differences between experimental groups. A value of *p* < 0.05 was considered as statistically significant.

## Results

### Reprogramming of MSC

**Fig. 1:**
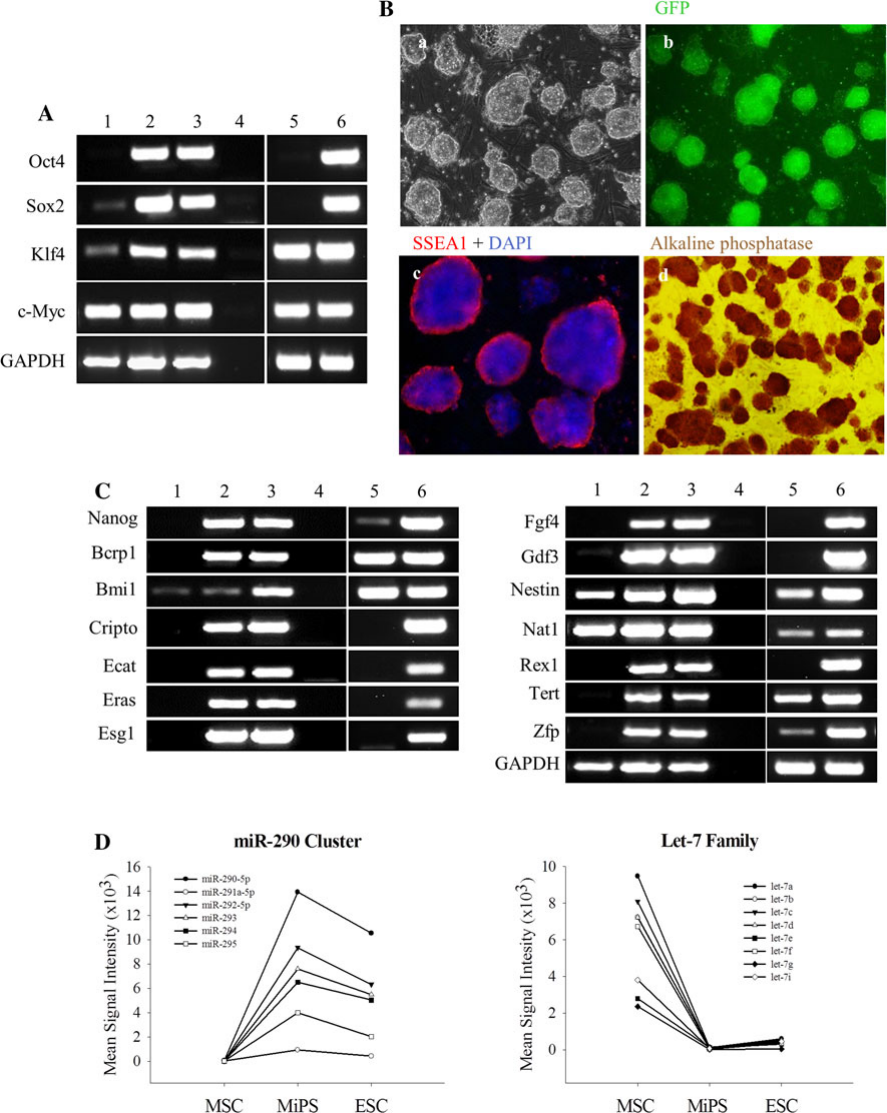
Overexpression of the four stemness factors led to reprogramming of MSC. **A** RT-PCR for endogenous expression of stemness factors in native MSC (*lane-1* MSC, *lane-2* MiPS clone, *lane-3* mouse ESC, *lane-4* negative control, *lane-5* MEF, *lane-6* FiPS). **B** 18–21 days after four-factor transduction, a typical ESC-like morphology was observed in MiPS clones; **a** phase-contrast and **b** fluorescent images of Oct4/GFP + MiPS colonies. **c** Fluorescence immunostaining of MiPS for SSEA-1 (*red*). Nuclei were visualized by DAPI staining (*blue*). **d** MiPS stained positive for alkaline phosphatase (*dark red*). **C** RT-PCR for ESC-specific gene expression in MiPS (*lane-1* MSC, *lane-2* MiPS clone, *lane-3* mouse ESC, *lane-4* negative control, *lane-5* MEF, *lane-6* FiPS). GAPDH was used as a loading control. **D** Relative miR expression for pluripotency and differentiation-related miRs, respectively during reprogramming of MSC. (Original magnifications: **a** and **b** ×20, **c** 20 × 1.6, **d** ×10)

Native MSC were isolated from Oct4-GFP transgenic mice and characterized by flow cytometry for MSC-specific markers (Supplementary Figure-I). The cell population used for reprogramming was 88.3 and 86.6 % pure for CD44 and CD29, respectively whereas the cells were very low in the expression for hematopoietic markers including CD34 (1.1 %) and CD45 (1 %). Analysis for stemness markers by RT-PCR showed that native MSC endogenously expressed three of the four Yamanaka stemness factors, including Sox2, Klf4, and c-Myc (Fig. 1a). MSC were successfully reprogrammed into MiPS by simultaneous overexpression of stemness factors including Oct4, Sox2, Klf4, and c-Myc. The efficiency of the reprogramming was 0.01 %. Reprogrammed MSC were similar to ESC in morphology and formed round, compact colonies with tight margins and clearly defined edges as early as 18 days post-transduction (Fig. 1b panel a). Furthermore, successful reprogramming of MSC was confirmed by the newly formed MiPS colonies expressing GFP (green fluorescence) as the cells were expressing endogenous Oct4-GFP (Fig. 1b panel b). Similar to fibroblast-derived iPSC (FiPS), endogenous levels of the stemness factors Oct4, Sox2, Klf4, and c-Myc were increased in MiPS besides expression of other pluripotency gene markers such as Nanog, ESC-specific gene 1 (Esg1), fibroblast growth factor 4 (Fgf4), growth and differentiation factor 3 (Gdf3), and telomerase reverse transcriptase (TERT) (Fig. 1c). On the other hand, exogenous transgene expression of the four stemness factors was diminished in MiPS thus suggesting their fully reprogrammed state (Supplementary Figure-IIA). Percent methylation of Oct4 and Nanog promoters was significantly reduced in MiPS (4.1 ± 0.4 %; 10.5 ± 2.7 %) and FiPS (5.0 ± 0.9 %; 6.9 ± 0.8 %) vs. native MSC (63.9 ± 9.1 %; 43.9 ± 15.9 %) and MEF (58.6 ± 6.3 %; 53.2 ± 11.0 %), which confirmed the successful reprogramming of the respective somatic cell type to pluripotent status (Supplementary Figure-IIB). Using Oct4 and Nanog promoter methylation status in various cell types as a positive control, although the change was insignificant, we observed a decrease in percent methylation of Flk1 in MiPS (1.9 ± 0.6 %) and FiPS (2.2 ± 0.6 %) as compared to MSC (5.8 ± 1.0 %) and MEF (3.9 ± 0.6 %), respectively (Supplementary Figure-IIB). A comparison of MSC and MiPS were positive for ESC-specific markers SSEA-1, Oct4, and Sox2 (Fig. 1b panel c and Supplementary Figure-IIC). MiPS also displayed a high level of alkaline phosphatase activity similar to ESC (Fig. 1b panel d). During induction of MSC to a pluripotent state, MiPS miR profile was altered resembling more of a ESC-specific miR expression pattern. Specifically, during the transition of MSC to a pluripotent status, the ESC-specific miR-290-295 cluster was upregulated in MiPS (Fig. 1d) and unlike native MSC, the differentiation-related let-7 family was almost abrogated in MiPS comparable to let-7 expression levels in ESC (Fig. 1d). Furthermore, DNA methyltransferase activity in MiPS was insignificantly different from ESC (Supplementary Figure-IIIA). Karyotyping showed that more than 70 % of MiPS clones were euploid (Supplementary Figure-IIIB). Pluripotency of MiPS was confirmed by teratogenicity subsequent to subcutaneous injection of the cells into immunodeficient nude mice (*n* = 3; Supplementary Figure-IV).

### In vitro cardiogenic differentiation of MiPS

**Fig. 2:**
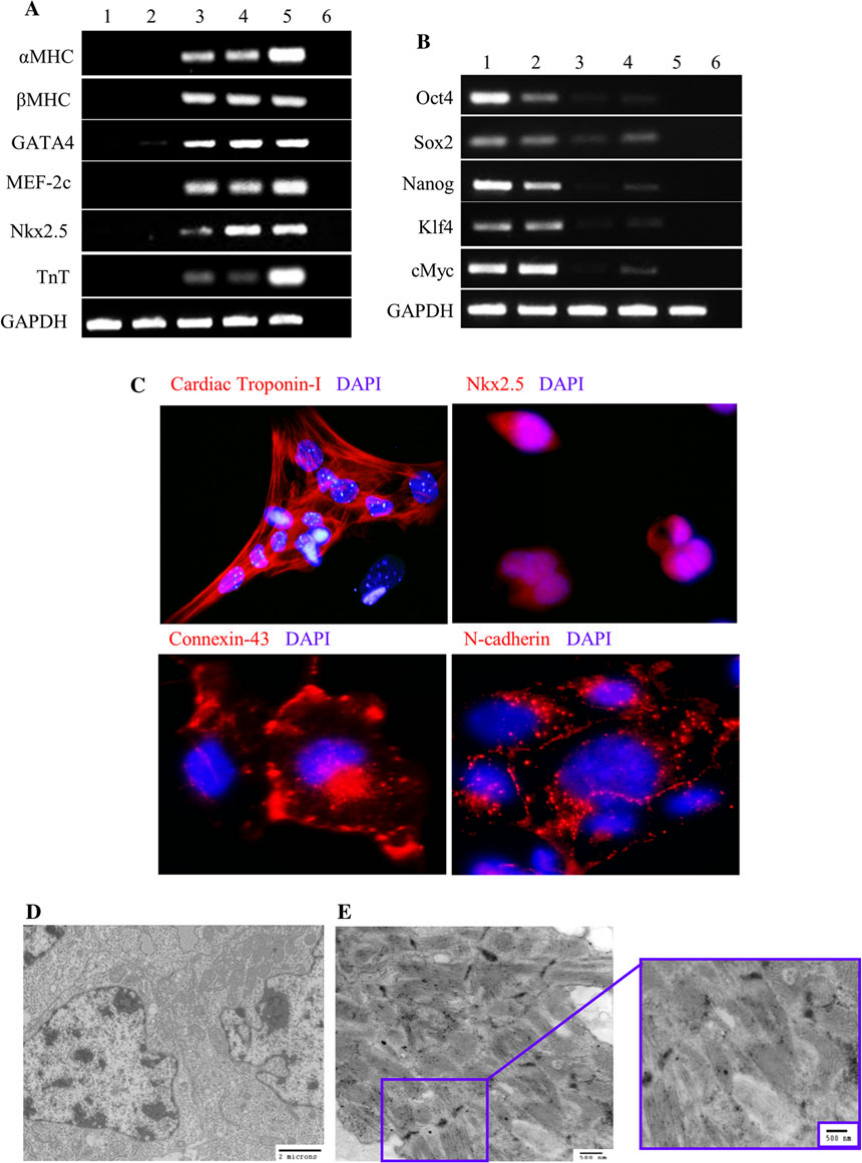
Spontaneous cardiac differentiation of MiPS into beating cardiomyocyte-like cells (MiPS-CP). **a** RT-PCR for cardiomyocyte-specific genes in MiPS-CP on day ten after EB formation (*lane-1* MiPS, *lane-2* early non-beating day-6 EBs, *lanes 3–4* MiPS-CP, *lane-5* the heart, *lane-6* negative control). **b** Pluripotency specific gene expression was down-regulated during spontaneous differentiation in vitro in parallel with increased expression of cardiac specific genes (*lane-1* MiPS, *lane-2* early non-beating day-6 EBs, *lanes 3–4* MiPS-CP, *lane-5* the heart, *lane-6* negative control). **c** Fluorescence immunostaining of MiPS-CP for cardiac-specific markers including cardiac troponin-I (*red*), Nkx2.5 (*red*), and gap-junction proteins connexin-43 (*red*) and N-cadherin (*red*). Nuclei were stained with DAPI (*blue*) for visualization (original magnifications: Tn-I = ×100; Nkx2.5, Cx-43, N-cad = 60 × 1.6). **d**, **e** Transmission electron micrograph showing ultra-structure of **d** MiPS with high nucleus to cytoplasmic ratio and **e** beating regions of the cells generated by spontaneous differentiation displayed striated structure resembling developing cardiomyocytes with z-lines and sarcomeric organization (original magnifications: *D* = ×10,000; *E* = ×20,000)

Using the standard EB differentiation protocol, MiPS were successfully differentiated into cardiomyocyte-like cells. MiPS were allowed to form aggregates in suspension culture and after 3 days in differentiation culture medium, EBs were transferred to adherent culture dishes. After culture for another 5–7 days, spontaneously beating regions of cells were observed (Supplementary Video-1). The beating CP derived from MiPS (MiPS-CP) were positive for cardiac-specific transcription factors and myogenic markers including GATA4, myocyte enhancer factor-2c (MEF2c), Nkx2.5, myosin heavy chain (αMHC and βMHC) and cardiac troponin-T (Fig. 2a). Mouse heart tissue was used as a positive control. Furthermore, gene expression of pluripotency markers Oct4, Sox2, Nanog, Klf4, and c-Myc was downregulated during MiPS differentiation into MiPS-CP (Fig. 2b). Fluorescence immunostaining confirmed that MiPS-CP expressed cardiac markers including cardiac troponin-I, Nkx2.5, and gap junction proteins connexin-43 and N-cadherin (Fig. 2c). Analysis of undifferentiated MiPS by transmission electron microscopy revealed a high nucleus to cytoplasm ratio (Fig. 2d). On the other hand, cells isolated from the spontaneously beating EBs contained highly organized striated fibers indicative of their spontaneous differentiation into beating cardiomyocytes (Fig. 2e).

### miR expression profiling of MiPS and MiPS-CP

**Fig. 3:**
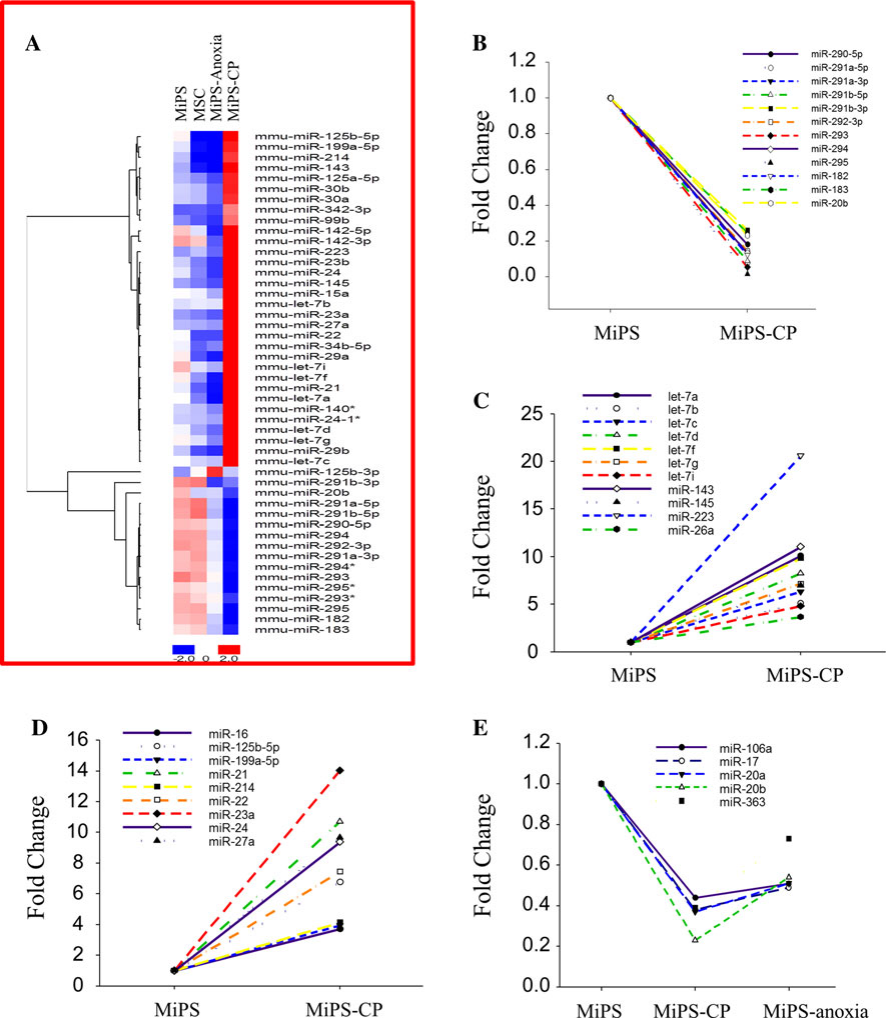
miR profiling of MiPS during differentiation into cardiac progenitors. **a** Heat map of miR expression profile in MSC, MiPS, MiPS-anoxia treated cells, and MiPS-CP. Red and blue colors denote miRs with upregulated and downregulated expression respectively. **b**, **c** Fold change in the embryonic stem cell specific miR-290 cluster and differentiation associated miRs including the let-7 family during cardiac differentiation of MiPS. **d** Expression profile of tumor suppressor miRs in MiPS-CP. **e** Fold changes in angiogenesis regulating miRs showed a similar trend in MiPS-CP as in anoxia-treated MiPS (MiPS-anoxia)

miR expression profiles revealed changes in miR expression during cardiac differentiation of MiPS (Fig. 3a). During MiPS differentiation into MiPS-CP, the embryonic-associated miR-290–295 cluster was significantly downregulated (Fig. 3b). Conversely, there was a significant increase in expression of differentiation-related miRs, including the let-7 family as well as miRs that were expressed in cardiac progenitors and regulated muscle differentiation (Fig. 3c). Differentiation of MiPS into cardiac progenitors also resulted in upregulation of tumor suppressor miRs including miR-125b, miR-145, miR-16, miR-199a, miR-21, miR-22, and miR-23a-24-27a cluster (Fig. 3d). We also observed changes in miRs that regulated angiogenic growth factors such as VEGF. MiPS subjected to anoxia showed downregulation of miR-20a and miR-20b, miRs which suppress VEGF expression (Fig. 3e).

### MiPS and derivatives attenuated infarction size expansion

**Fig. 4:**
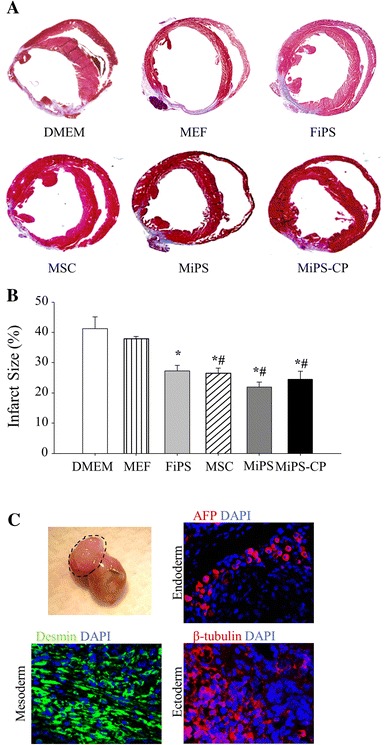
Transplantation of MiPS-CP reduced infarction size. **a**, **b** Masson’s trichrome staining 4 weeks after permanent LAD ligation showed attenuated infarction size in various treatment groups of animals (*n* = 4/group). Infarct size was significantly attenuated in cell transplanted animal groups (MSC treated group-1, MiPS and MiPS-CP treated groups-2 and 3, and FiPS group-4 * *p* < 0.001 vs. control). MSC, MiPS, and MiPS-CP treatment groups also had a significantly reduced infarction size compared to MEF group-5 (^*#*^ *p* < 0.01). All values were expressed as mean ± SEM. **c** Tumor found in undifferentiated MiPS transplanted heart 4 weeks after treatment. The tumor expressed three germ layer markers including alpha-fetoprotein (*red*), desmin (*green*), and beta-tubulin (*red*). Nuclei were stained with DAPI (original magnification = ×40)

To determine cardiac reparability of MiPS and MiPS-CP, the cells were transplanted into the heart in an immunocompetent mouse model of myocardial infarction. After 4 weeks of respective treatments, histological studies (*n* = 4/group), showed the damaged area of the LV wall which was evident from formation of scar tissue (Fig. 4a). However, the cell transplanted groups including MSC (26.6 ± 1.6 %), MiPS (21.9 ± 1.7 %), MiPS-CP (24.6 ± 2.7 %), and FiPS (27.3 ± 1.9 %) treatment groups had attenuated infarction size and reduced LV wall thinning (41.3 ± 3.8 %; *p* < 0.001 vs. DMEM group; Fig. 4b). Moreover, MSC, MiPS, and MiPS-CP had significantly reduced infarction size compared to MEF group (38.0 ± 0.8 %; *p* < 0.01).

Consistent with our previous report that transplantation of undifferentiated iPSC formed cardiac tumors in immunocompetent hosts [1], we observed cardiac tumors in 21 % of undifferentiated MiPS transplanted animals (Fig. 4c). Immunohistological studies confirmed that the cardiac tumors stained positive for cell surface markers specific for all the three germ layers including endoderm (α-fetoprotein), mesoderm (desmin), and ectoderm (β-tubulin) (Fig. 4c and Supplementary Figure-V). No teratomas were observed in native MSC and MiPS-CP transplanted animal hearts.

### Pro-angiogenic effects of MiPS and MiPS-CP in the infarcted heart

**Fig. 5:**
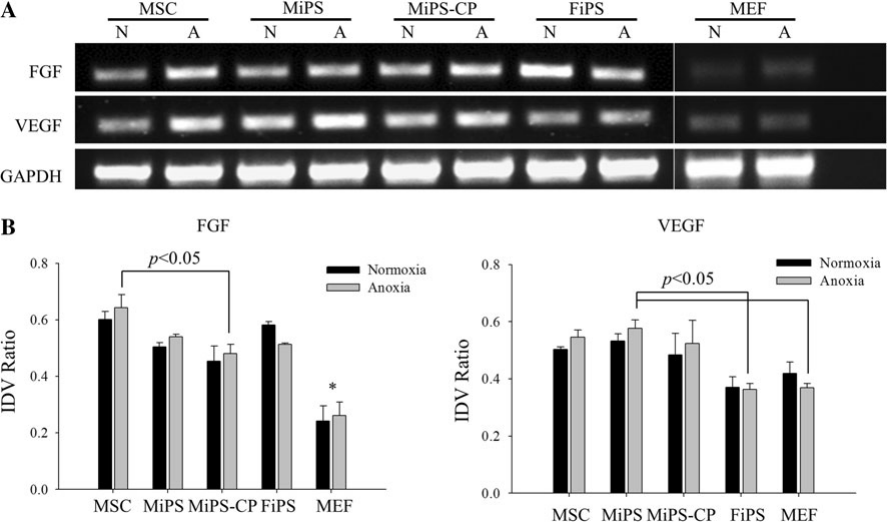
Paracrine behavior of MiPS and MiPS-CP is similar to native MSC. **a** RT-PCR for angiogenic growth factors in different cell groups under normoxia (N) and anoxic conditions (A). Expression of VEGF was less in MEFs and FiPS compared to the other groups and although FiPS did express FGF, there was a decrease in expression after anoxia treatment, whereas there was an observed increase in FGF in MSCs, MiPS and MiPS-CP after anoxia. **b** Quantitative densitometry for angiogenic gene expression with GAPDH used as loading control (* *p* < 0.05 MEF normoxia and anoxia vs. all other groups; *n* = 3)

**Fig. 6:**
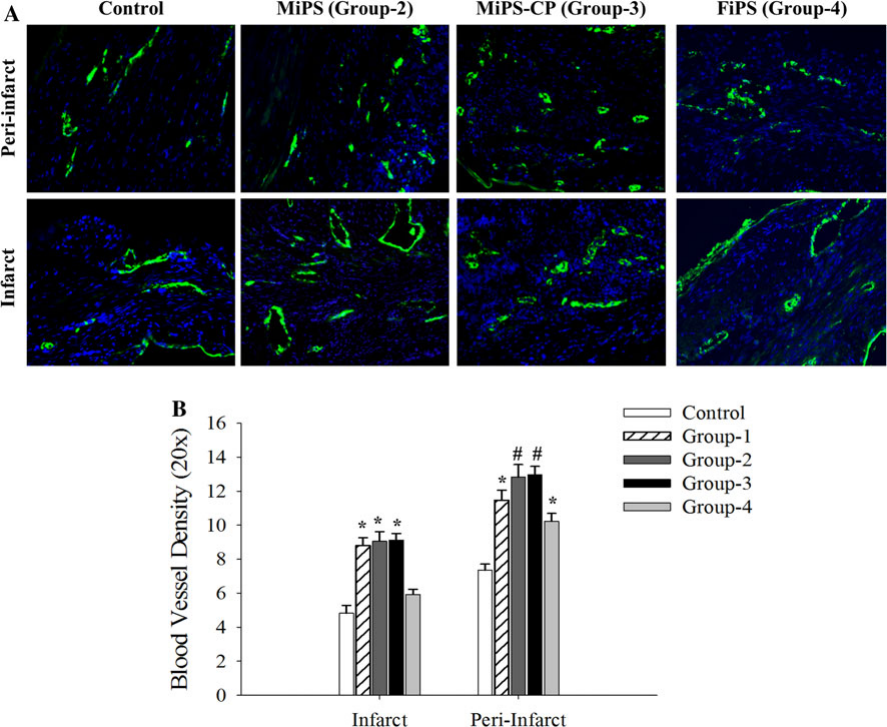
MiPS and MiPS-CP improved angiogenesis in the ischemic heart. **a** Immunofluorescence staining of mouse heart tissue sections from different treatment groups for von Willebrand factor-VIII at 4 weeks after their respective treatment. **b** Quantitative analysis confirmed that MSC, MiPS, and MiPS-CP transplanted groups-1, 2, and 3 showed significantly higher blood vessel density than both DMEM controls and FiPS group-4 in the infarct region (* *p* < 0.001). In peri-infarcted areas, MiPS and MiPS-CP showed significantly improved density compared to DMEM control and FiPS groups (^#^ *p* < 0.01) while both MSC and FiPS were better than DMEM group (* *p* < 0.01). All values were expressed as mean ± SEM

We observed that native MSC expressed VEGF and FGF under normal conditions which increased under anoxic culture conditions (Fig. 5). Similar to native MSC, both MiPS and MiPS-CP, as well as FiPS, expressed basal levels of VEGF and FGF that were increased after anoxia. We observed only low levels of VEGF and FGF expression in anoxia treated MEF (Fig. 5) Consistent with these data, significantly increased blood vessel density was observed in the animal hearts 4 weeks after transplantation of native MSC (8.8 ± 0.5; 11.5 ± 0.6), MiPS (9.1 ± 0.6; 12.8 ± 0.7), and MiPS-CP (9.1 ± 0.4; 12.9 ± 0.5) in the infarct and peri-infarct regions, respectively compared to control animals (4.8 ± 0.5; 7.4 ± 0.4; *p* < 0.001) (Fig. 6). Blood vessel density was also improved in FiPS (5.9 ± 0.3; 10.2 ± 0.5) transplanted animals in the peri-infarct region (*p* < 0.01 vs. DMEM group). However, blood vessel density was significantly higher in MSC, MiPS, and MiPS-CP groups as compared to FiPS in the infarct region and in MiPS and MiPS-CP as compared to FiPS in the peri-infarct regions (*p* < 0.01).

### MiPS-CP differentiated into cardiomyocytes in the infarcted heart

**Fig. 7:**
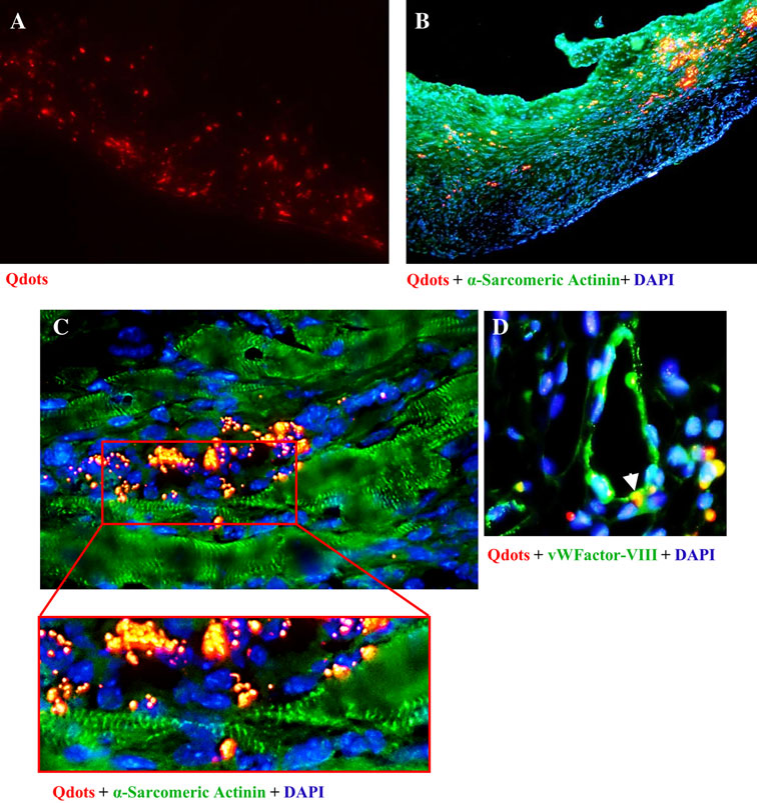
Transplanted MiPS-CP adopted angiomyogenic fate post-transplantation. **a** Histological sections showing extensive presence of Qdot-labeled MiPS-CP in the infarcted heart 4 weeks after transplantation. **b**, **c** Immunostaining of MiPS-CP transplanted hearts after 4 weeks indicated co-localization of transplanted MiPS-CP (*red*) and the myogenic marker cardiac α-sarcomeric actinin (*green*). The red box in C indicates the area that was magnified (*below*) to show the transplanted MiPS-CP (*red*) overlapping with α-sarcomeric actinin (*green*) in the infarcted region of the myocardium. **d** Immunostaining for the endothelial marker von Willebrand factor-VIII showed co-localization of transplanted MiPS-CP (*red*) with vWF-VIII (*green*). Nuclei were stained with DAPI (original magnifications: **a**, **b** = ×20; **c** = ×40; **d** = ×100)

Extensive presence of Qdot (red fluorescence) labeled MiPS-CP was observed at the site of the cell graft 4 weeks after transplantation (Fig. 7a). Immunohistochemistry revealed co-localization of myogenic marker α-sarcomeric actinin (green) with red fluorescence in the infarct (Fig. 7b) and peri-infarct (Fig. 7c) areas of the MiPS-CP transplanted hearts. Similarly, MiPS-CPs were also co-localized with the angiogenic marker von Willebrand factor-VIII (green) thus suggesting that several of the transplanted cells were also incorporated into blood vessels (Fig. 7d).

### Heart function studies

**Fig. 8:**
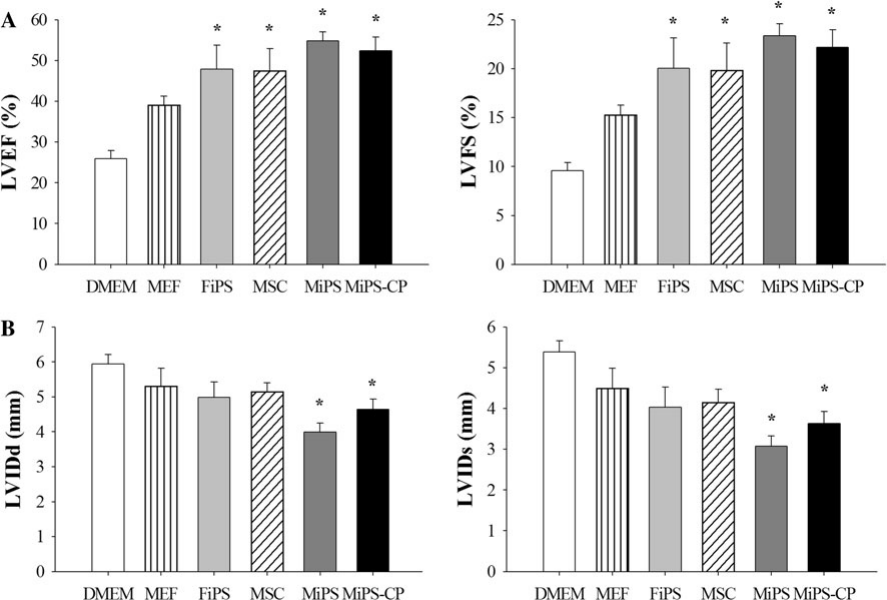
Transplantation of MiPS and MiPS-CP preserved global heart function. **a** Heart function was determined by echocardiography 4 weeks after myocardial infarction and after appropriate cell treatments. Treatment with MEFs did not significantly change heart function. LVEF and LVFS were significantly improved in all other cell transplanted groups as compared to basal DMEM injected control (* *p* < 0.001). Although there was a significant improvement in ejection fraction in MiPS and MiPS-CP treated groups-2 and 3, there were no significant differences between the cell transplanted groups. **b** The DMEM-injected control animals also showed worsened LV chamber dimensions 4 weeks post-infarction, whereas we observed significantly preserved LV chamber dimensions during diastole (LVIDd: * *p* < 0.01 vs. DMEM) and systole (LVIDs: * *p* < 0.01 vs. DMEM) in both MiPS and MiPS-CP treated groups-2 and 3. All values were expressed as mean ± SEM. (*n* = 7; MSC *n* = 6; FiPS *n* = 5; MEF *n* = 3)

Four weeks after permanent LAD artery ligation, indices of LV contractile function including LV ejection fraction (LVEF) and fractional shortening (LVFS) were significantly deteriorated in all the treatment groups (*n* = 7) as compared to baseline controls (*n* = 4; 79.4 ± 2.6 %; 41.3 ± 2.4 % respectively, *p* < 0.001 vs. all cell transplanted groups). On the contrary, cell transplantation led to significant improvement in global heart function (Fig. 8a). As compared to DMEM group (25.9 ± 2.1 %; 9.6 ± 0.8 %), LVEF and LVFS were significantly improved in native MSC (47.4 ± 5.5 %; 19.8 ± 2.8 %), MiPS (54.8 ± 2.2 %; 23.4 ± 1.2 %), and MiPS-CP treated animal hearts (52.4 ± 3.4 %; 22.2 ± 1.8 %). Furthermore, LVEF and LVFS in FiPS treatment group were significantly improved (47.9 ± 5.9 %; 20.0 ± 3.1 %, respectively) and remained insignificantly changed in MEF (39.1 ± 2.2 %; 15.3 ± 1.0 %) as compared to DMEM group. Cell transplantation also prevented pathological remodeling of the LV (Fig. 8b). LV chamber dimensions during systole (LVIDs) and diastole (LVIDd) were significantly improved (*p* < 0.01) in both MiPS (3.1 ± 0.3 mm; 4.0 ± 0.3 mm, respectively) and MiPS-CP (3.6 ± 0.3 mm; 4.6 ± 0.3 mm) treated groups compared to DMEM controls (5.4 ± 0.3 mm; 6.0 ± 0.3 mm). However, no significant difference was observed in LVIDs or LVIDd in FiPS (4.0 ± 0.5 mm; 5.0 ± 0.5 mm) and MEF (4.5 ± 0.5 mm; 5.3 ± 0.5 mm) treated groups as compared to DMEM group.

## Discussion

The breakthrough discovery of somatic cell reprogramming is a promising approach for the generation of autologous, pluripotent stem cells for use in regenerative medicine. Nevertheless, a few studies have focused on myocardial reparability of iPSC [2, 20]. Moreover, these studies have published conflicting results in terms of the teratogenicity of the transplanted cells. Therefore, both safety and efficacy of iPSC warrant in-depth and systematic assessment in experimental animal models. We reprogrammed MSC using Yamanaka’s conventional four-factor protocol [34] and determined the safety and efficacy of MiPS and their derived MiPS-CP for myocardial repair. The important findings of our study included: (1) Successful and efficient reprogramming of MSC with four stemness factors in MSC. (2) MiPS resembled ESC in pluripotency gene and miR expression profiles and showed spontaneous cardiogenesis. (3) Transplantation of MiPS led to teratoma formation in the infarcted hearts of immunocompetent hosts. (4) Transplantation of MiPS-CP was safe and preserved infarcted heart function via angiomyogenic differentiation.

MSC constitute a heterogeneous group of cells some of which also express primitive surface markers. Our RT-PCR results confirmed that the cells used for reprogramming expressed Sox2, Klf4 and c-Myc besides showing low Oct4 expression. Irrespective of the strategy and type of the somatic cell used for reprogramming, iPSC display ESC-like traits albeit with varying degree of reprogramming efficiency and epigenetic characteristics which are related to the parent cell [9]. Keratinocytes, having higher endogenous Klf4 and c-Myc expression than fibroblasts, were reprogrammed after only 10 days of viral transgene expression as compared to weeks for complete reprogramming of fibroblasts [16]. Embryonic tissues in this regard, provide the most ideal parent cell population due to ease of reprogramming albeit for availability and ethical issues associated with their use [18]. It is now becoming more evident that iPSC retain the epigenetic memory of their parent cells due to incomplete DNA methylation patterns [8, 13, 23]. During transcription factor-based reprogramming strategy, DNA demethylation occurs at a slow rate and therefore, residual methylation can contribute to preservation of parent cell methylation signatures [8]. Epigenetic differences between ESC and iPSC contribute to altered differentiation potential in iPSC. It is therefore critical to consider the type of parent cell used based on tissue type and differentiation status [12, 13]. Comparison of cardiogenic differentiation potential of human iPSC derived from BM cells, skin fibroblasts, and hair keratinocytes demonstrated that there was no significant difference in functionality of the cardiomyocytes derived from each of the iPSC lines; however, BM-derived iPSC displayed significantly higher cardiac differentiation efficiency than fibroblast and keratinocyte-derived iPSC [33]. Using Flk1 as a model, we did not detect significant differences in percent methylation between native MSCs vs. MEFs. We also observed that percent methylation was similar in MiPS vs. FiPS. Furthermore, the change in percent methylation of Flk1 was not significant after reprogramming of MSC and MEF. Future in-depth and expansive studies would be required for more conclusive evidence to infer the role of parent cell epigenetics as a determinant of iPSC characteristics.

Given the autologous availability, multipotentiality and presence of subpopulations expressing stemness factors including Oct4, Nanog, alkaline phosphatase and SSEA-4 [28], we hypothesized that MSC provided a near optimal source of cells for reprogramming to pluripotency as compared to the conventionally used fibroblasts. Moreover, having epigenetic memory of MSC, MiPS would be more suitable for cardiac repair than fibroblasts as they can regenerate muscle fibers in addition to improving angiogenesis in the ischemic heart. Although our results demonstrated no statistical significance in the efficacy of MSC in comparison to its derivative MiPS and MiPS-CP, the latter two showed higher and better preserved LVEF and LVFS as well as LV diastolic and systolic dimensions as compared to MSC treated animal hearts. Similarly, both MiPS and MiPS-CP were as good as MSC in their response to anoxia to release angiogenic growth factors in vitro and enhanced angiogenesis in the heart. Based on these data, MiPS-CP has advantage of being committed to cardiogenic differentiation and therefore would be a better choice for transplantation. Similarly, differentiation into cardiac-like cells ensures tumor-free integration into the host myocardium. However, further in-depth experimentation in larger animal group size would be required for comprehensive comparison of MSC with its derivative reprogrammed cells.

The capacity of BM stem cells to cross lineage restriction and adopt myogenic as well as vasculogenic phenotypes after transplantation has been reported [10, 25, 29, 32]. However, limited cardiogenesis with BM stem cells has always been a concern [14, 19]. Alternatively, secretion of bioactive proangiogenic molecules, i.e. VEGF and FGF, integral to paracrine activity of stem cells has been implicated in cardioprotection either by pro-survival signal transduction or by creating a favorable concentration gradient in the heart to engage resident stem/progenitor cells for angiomyogenic repair. The present study, however, was more focused on myocardial reparability of MiPS and MiPS-CP and hence did not investigate the role of endogenous cardiac progenitors in myocardial repair process. Moreover, the use of genetic markers would be a more appropriate choice in order to determine the angiogmyogenic fate of the transplanted cells.

Undifferentiated iPSC, irrespective of their origin and method of reprogramming, are inherently teratogenic post-transplantation similar to undifferentiated ESC [21]. We have already shown that transplantation of skeletal myoblast derived iPSC formed tumors in the hearts of immunocompetent hosts [1] unlike the published data [20]. During the present study, similar rate of teratogenicity was observed in the undifferentiated MiPS transplanted animal hearts. However, in agreement with our previous study, we did not observe teratomas in the hearts transplanted with differentiated MiPS-CP isolated from spontaneously differentiated EBs [2]. These data suggested that undifferentiated iPSC were similar to ESC in tumorigenic potential and therefore should be used with caution. Various strategies are being developed ranging from optimization of iPSC generation protocols to post-reprogramming manipulation of reprogrammed cells prior to transplantation in order to curtail their tumorigenic potential. For example, purification of cardiac progenitors based on surface markers and their differentiation to cardiac-like cells may enhance their cardiogenic potential without tumorigenicity [42]. More recently, flow cytometry assisted isolation method based on DNA contents and mitochondrial propensity have been developed to separate non-tumorigenic ESC sub-lineages [5]. In the same context, cardiac-like cells have been isolated with Percoll density gradient taking advantage of the size difference between ESC and their derived myocytes [39, 41]. Although differentiation of MiPS to MiPS-CP reduced the incidence of teratoma formation in the heart, mechanical dissection approach to isolate beating cardiomyocyte-like cells from the culture has its limitations and may not be an ideal strategy to ensure tumor-free use of iPSC.

With emergence of miRs as regulators of stem cell pluripotency and differentiation potential [24], we observed altered miR expression during reprogramming and differentiation to form MiPS and MiPS-CP respectively. We observed significant changes in miR-290 and let-7 families which were determinant of pluripotent and differentiated status of the cells, respectively. The miR-290 family are known ESC-specific cell cycle-regulating miRs that help to ensure ESC differentiation when required. With repression of Oct4, Sox2 and Nanog during spontaneous differentiation of MiPS, a significant abrogation of miR-290-295 and five to ten-fold increase in let-7 cluster was observed. On a similar note, miR-143 and miR-145 that regulate smooth muscle differentiation [40] as well as the muscle-specific miR-26a [37] were also increased. These data suggested that MiPS-CP expressed miRs which were directly involved in muscle and vascular cell differentiation. A comparison of miR expression profile showed that differentiation of MiPS prior to transplantation increased tumor-suppressor miRs thus reducing the chances of tumorigenicity. We observed upregulated expression of cell growth specific miRs including miR-125b, miR-145, miR-16 and miR-199a during differentiation of MiPS into MiPS-CP [31, 35]. MiPS-CP also showed increased expression of miR-21, miR-22, and miR-23a-24-27a cluster that function to inhibit cell growth and proliferation either through suppression of Oct4, Sox2, and Nanog or by targeting c-Myc [4, 17]. Interestingly, we also observed decreased levels of miR-20a-b, the members of miR-17 family expressed in many cancers and may contribute to tumor growth [3]. Both miR-20a-b target VEGF and under hypoxic conditions, they are significantly down-regulated in response to stabilization of hypoxia inducible factor-1α (HIF-1α) [3]. These findings suggested that differentiation of MiPS into cardiac progenitors not only upregulated genes and miRs involved in cardiac differentiation, but also helped to reduce tumorigenicity. In conclusion, differentiation of MiPS prior to transplantation is a safer option to achieve tumor-free use of MiPS. However, protocols need to be developed and optimized to ensure the purity of MiPS-CP and determination of the level of differentiation of MiPS towards cardiac commitment would ensure better prognosis.

## Electronic supplementary material

Below is the link to the electronic supplementary material.

Supplementary material 1 (TIFF 1254 kb)

Supplementary material 2 (TIFF 236 kb)

Supplementary material 3 (TIFF 717 kb)

Supplementary material 4 (TIFF 4412 kb)

Supplementary material 5 (TIFF 1444 kb)

Supplementary material 6 (TIFF 737 kb)

Supplementary material 7 (TIFF 286 kb)

Supplementary material 8 (DOC 55 kb)
