# Supplementary figures and images for: Cardiac progenitors derived from reprogrammed mesenchymal stem cells contribute to angiomyogenic repair of the infarcted heart
Source: Basic Res Cardiol. 2012 Oct 18;107(6):301. doi: 10.1007/s00395-012-0301-5 (PMC3505546; doi:10.1007/s00395-012-0301-5)

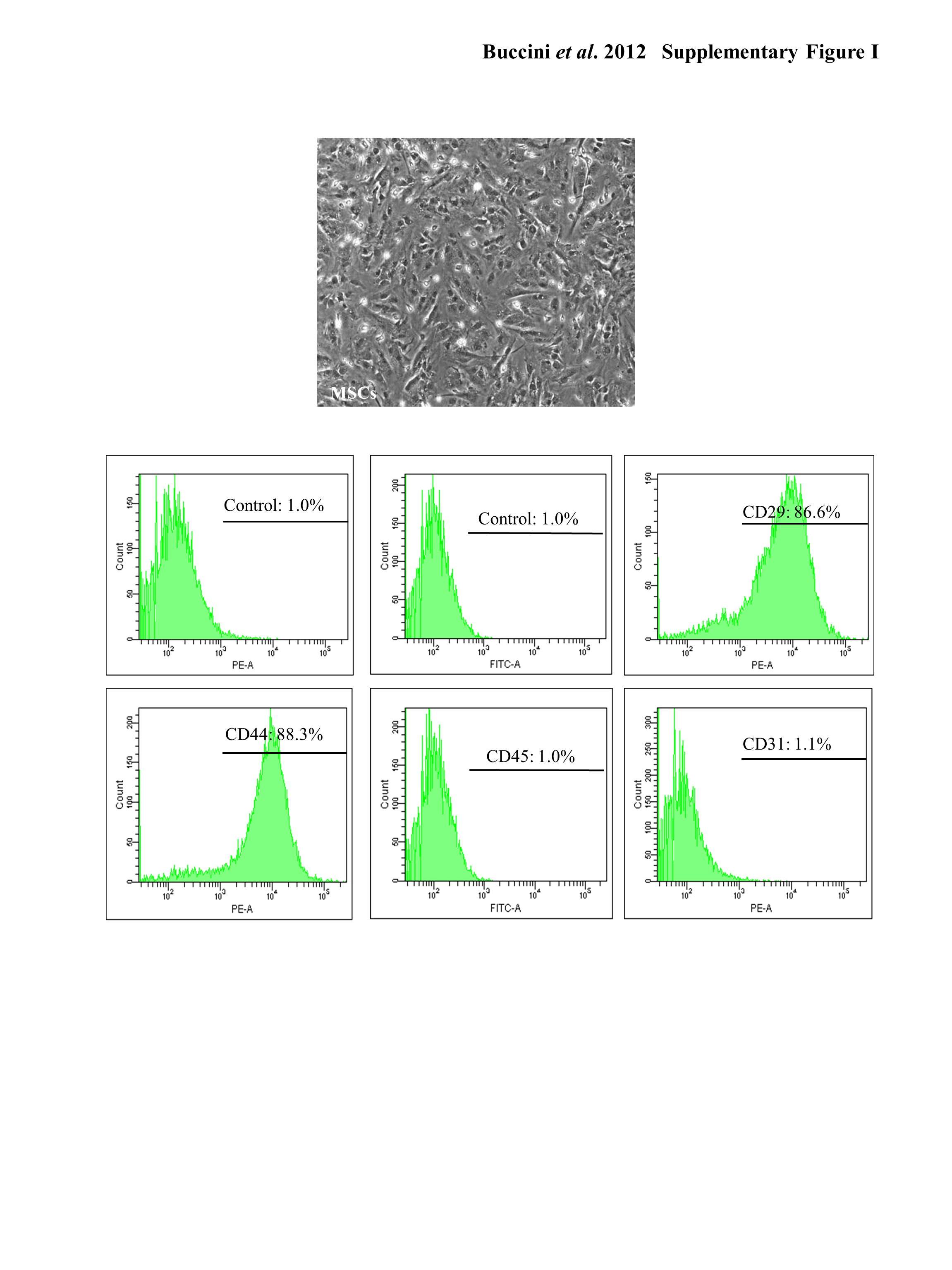

Supplement: Supplementary file 1 — Supplementary material 1 (TIFF 1254 kb) [file 395_2012_301_MOESM1_ESM.tif]

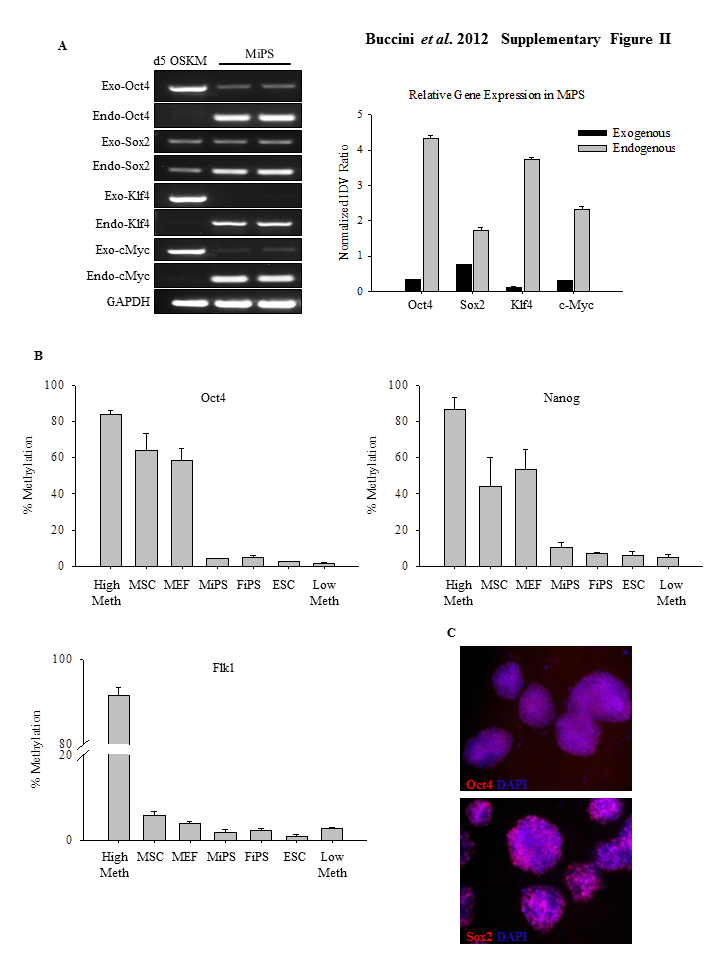

Supplement: Supplementary file 2 — Supplementary material 2 (TIFF 236 kb) [file 395_2012_301_MOESM2_ESM.tif]

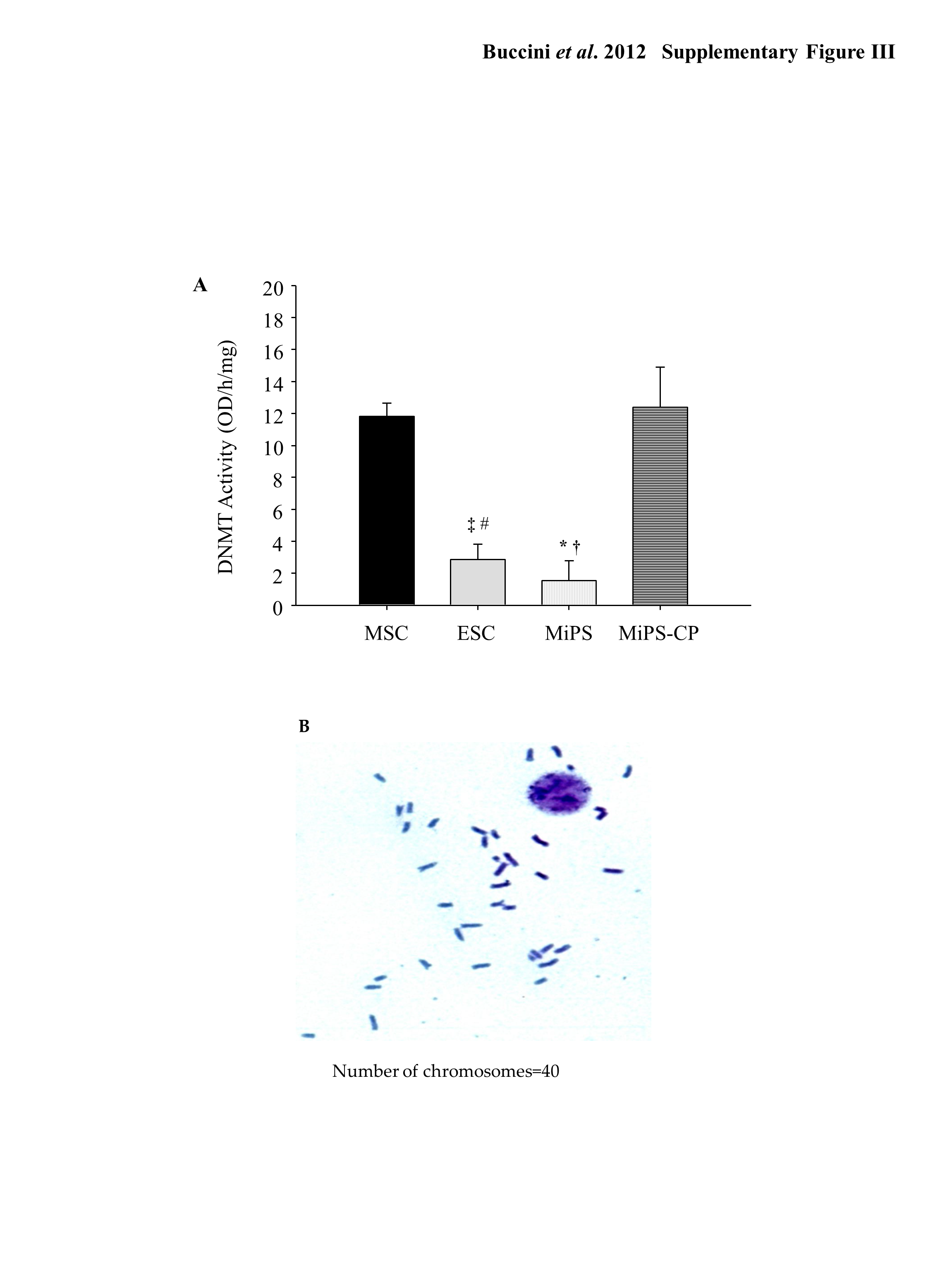

Supplement: Supplementary file 3 — Supplementary material 3 (TIFF 717 kb) [file 395_2012_301_MOESM3_ESM.tif]

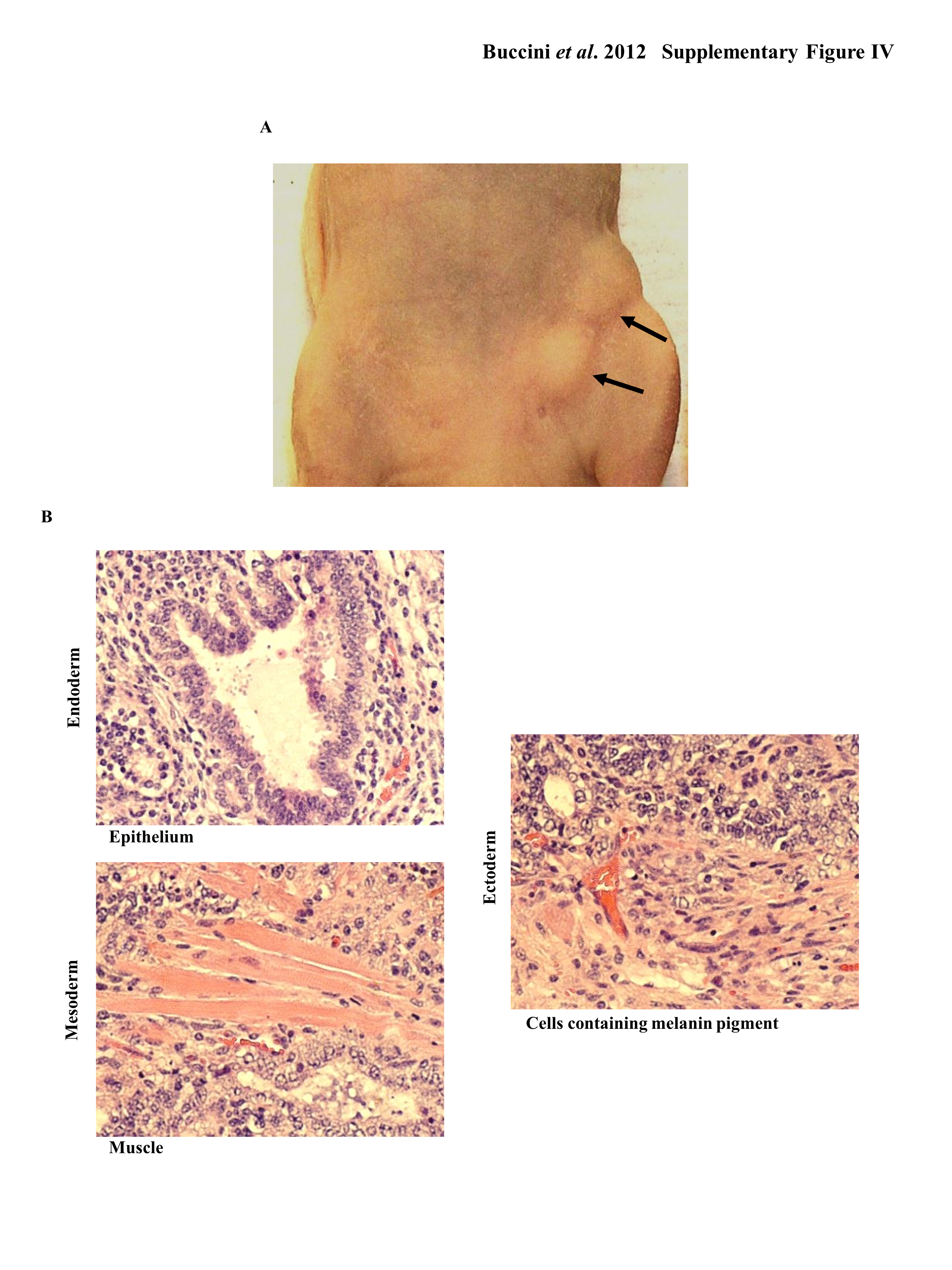

Supplement: Supplementary file 4 — Supplementary material 4 (TIFF 4412 kb) [file 395_2012_301_MOESM4_ESM.tif]

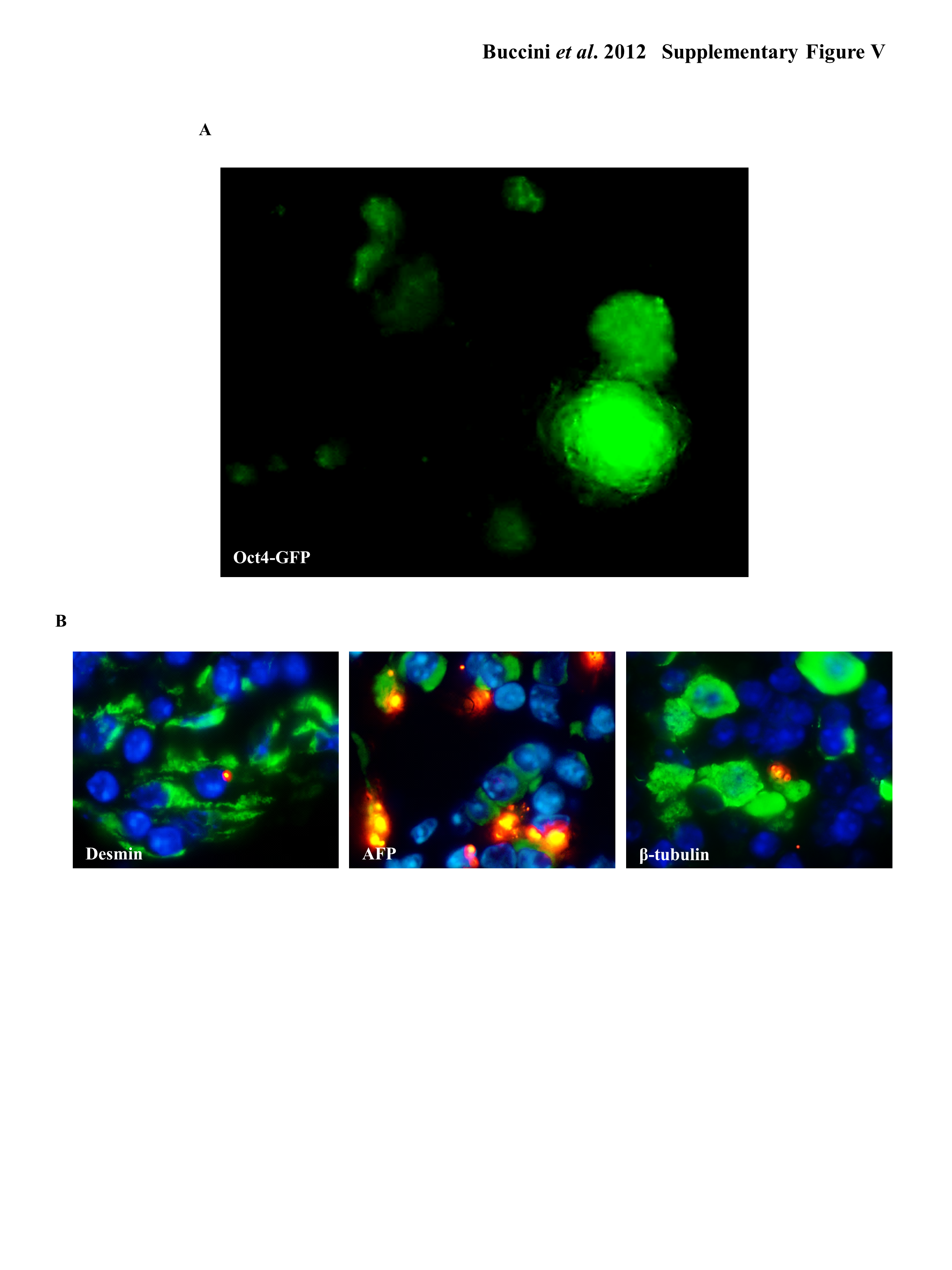

Supplement: Supplementary file 5 — Supplementary material 5 (TIFF 1444 kb) [file 395_2012_301_MOESM5_ESM.tif]

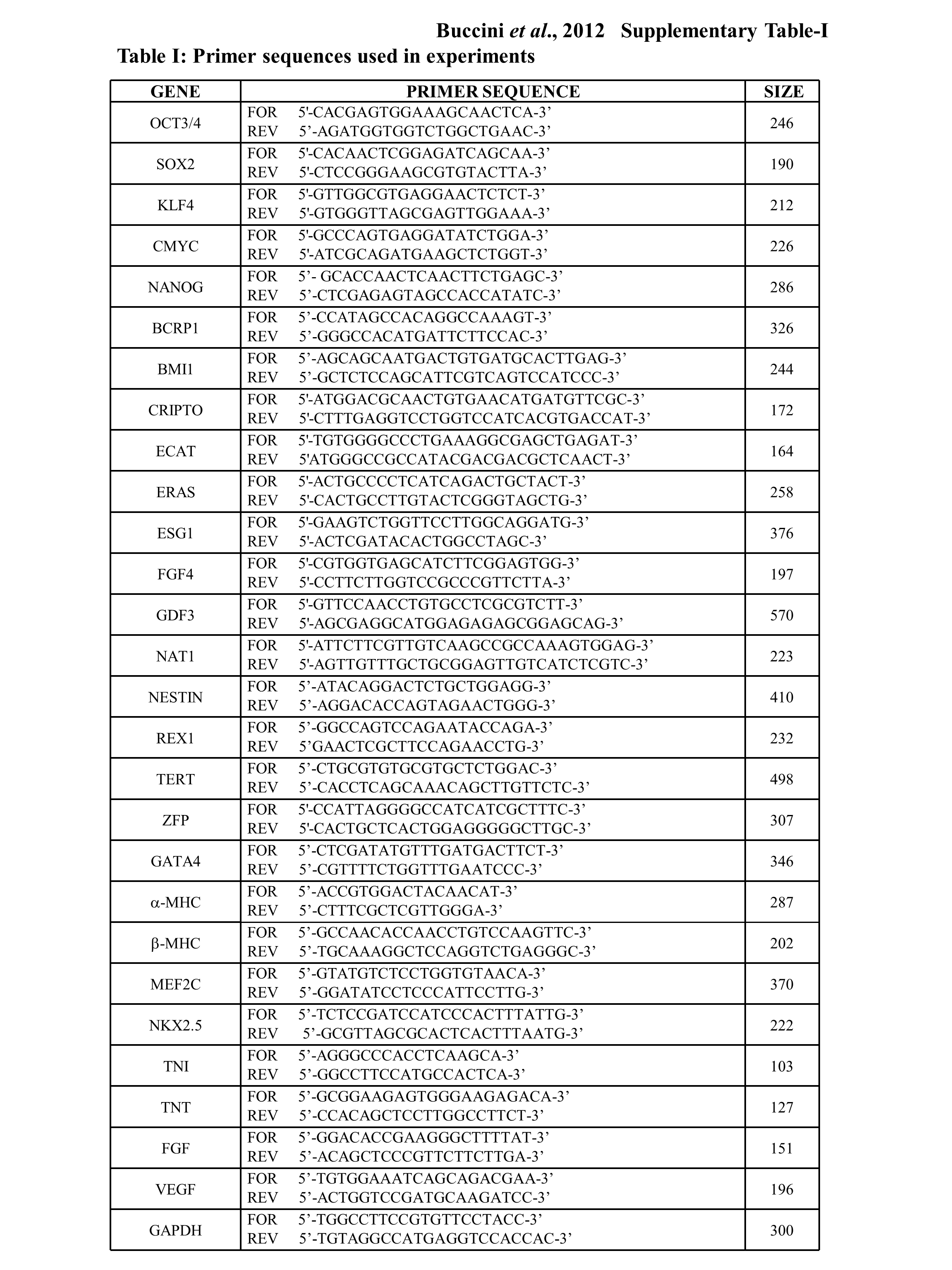

Supplement: Supplementary file 6 — Supplementary material 6 (TIFF 737 kb) [file 395_2012_301_MOESM6_ESM.tif]

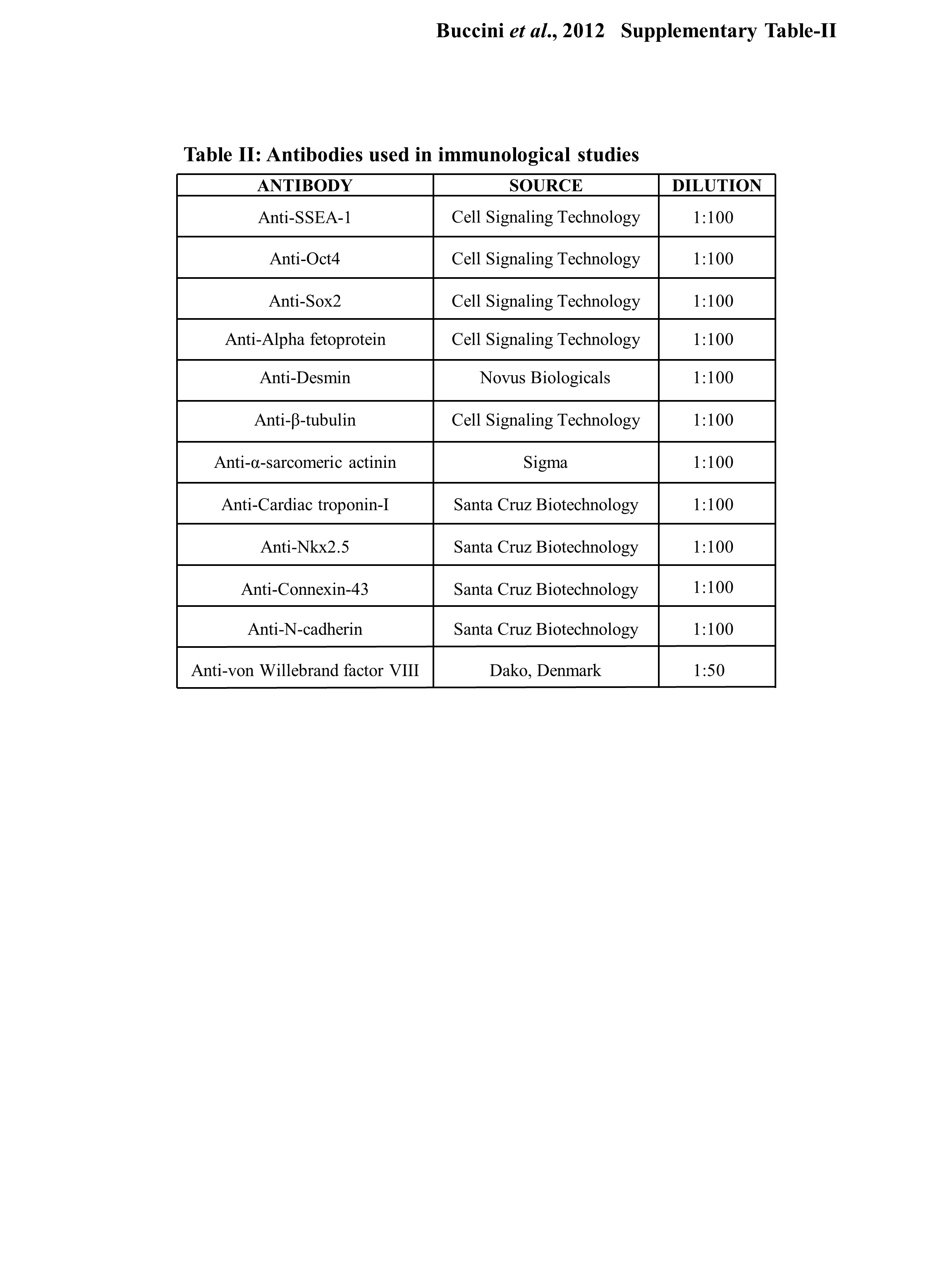

Supplement: Supplementary file 7 — Supplementary material 7 (TIFF 286 kb) [file 395_2012_301_MOESM7_ESM.tif]
