# Supplementary material for: Cardiac progenitors derived from reprogrammed mesenchymal stem cells contribute to angiomyogenic repair of the infarcted heart
Source: Basic Res Cardiol. 2012 Oct 18;107(6):301. doi: 10.1007/s00395-012-0301-5 (PMC3505546; doi:10.1007/s00395-012-0301-5)
Supplement: Supplementary file 8 — Supplementary material 8 (DOC 55 kb) [file 395_2012_301_MOESM8_ESM.doc]

**Supplementary Data**

**Supplementary Materials and Methods**

All experimental procedures performed were approved by the Institutional Animal Care and Use of Committee at the University of Cincinnati, which conformed to the Guidelines for the Care and Use of Laboratory Animals published by the National Institutes of Health.

**Isolation and characterization of MSC**

MSC were purified from young (4-6 weeks old) male, Oct4-GFP transgenic mice (Jackson Laboratories, ME, USA) with GFP tagged to the endogenous Oct4 gene promoter to monitor reprogramming as reprogrammed cells showed GFP fluorescence. For isolation of MSC, bones were isolated and washed first in 70% alcohol then DPBS and then flushed with high glucose Dulbecco’s modified Eagle’s medium (DMEM; 10% fetal bovine serum and 0.5% penicillin/streptomycin)^8^. The cells were centrifuged at 1200 rpm for 5 minutes to remove any tissue debris. The adherent MSCs were propagated and maintained in high glucose DMEM containing 10% fetal bovine serum (FBS) and 0.5% penicillin/streptomycin at 37˚C, 5% CO_2_ atmosphere. Fresh medium was added every 3 days. The cells were maintained and expanded for no more than 3 passages for subsequent transfections.

The adherent cells were harvested with 0.025% trypsin (Sigma Aldrich, MO, USA) and after washing with DPBS 5x10^5^ cells were incubated with 1 µg/ml PE-conjugated anti-mouse CD44 (BD BioScience, NJ, USA), CD29 (eBioSciences, CA, USA), PE-conjugated CD31 or FITC-conjugated anti-mouse CD45 (Beckman Coulter, NJ, USA)^4^. After washing twice with DPBS, cells were resuspended in 500 µL DPBS and analyzed by flow cytometry (BD FACSCanto, BD Biosciences, NJ, USA). Unlabeled cells were used as a control.

**RNA isolation and reverse transcription**

Total RNA was isolated using the RNeasy Mini Kit (Qiagen, CA, USA), according to the manufacturer’s instruction. One microgram total RNA was used for first-strand cDNA synthesis and genomic DNA removal, performed with the QuantiTect Reverse Transcription Kit (Qiagen), per manufacturer’s instructions. Reverse transcription polymerase chain reaction (RT-PCR) was done using Taq DNA Polymerase (Qiagen, CA, USA) and the products were run on an agarose gel^10^. Primer sequences are given in Supplementary Table-I.

**Alkaline phosphatase staining**

Alkaline phosphatase staining was carried out using the Alkaline Phosphatase Detection Kit (Millipore, CA, USA) per manufacturer’s instructions. Briefly, MiPS were cultured for 5 days and fixed with 4% paraformaldehyde for 1-2 minutes. The cells were stained with reagents provided in the kit in the dark at room temperature for 15 minutes before observation.

**Immunocytochemistry**

The cells were fixed with 4% paraformaldehyde in PBS for 20 minutes on ice, and after washing with PBS, the cells were treated with PBS containing 0.1% Triton X-100 for 5 minutes^11^. The cells were later incubated with CAS-BLOCK (Invitrogen, CA, USA) for 1 hour at room temperature and subsequently with the specific primary antibodies (Supplementary Table-II). The primary antigen-antibody reaction was detected using goat anti-rabbit Alexa Fluor-546 conjugated secondary antibody (1:200, Invitrogen, CA, USA) and goat anti-mouse Alexa Fluor-546 conjugated secondary antibody (1:200, Invitrogen, CA, USA).

**DNA methyltransferase activity assay**

Nuclear extract was isolated using NE-PER Nuclear and Cytoplasmic Extraction Kit (Thermo Scientific, MA, USA). Extracts were quantified with the DC Protein Assay Kit (Bio-Rad, CA, USA). DNA methyltransferase (DNMT) activity was measured using the EpiQuick DNA Methyltransferase Activity/Inhibition Assay Kit (Epigentek, NY, USA) per manufacturer’s instructions. Absorbances were read on a microplate reader at 450 nm.

**Teratoma formation and Karyotyping**

For teratoma formation assays, immunodeficient nude mice were purchased from Jackson Laboratories (Jackson Labs. ME, USA). Intramuscular injection of undifferentiated MiPS (2x10^5^) was performed in 2-3 month old nude mice. After 2 weeks teratomas were removed and fixed with 10% formalin for hematoxylin and eosin staining. For karyotyping, the cells were treated with 0.1µg/ml Colcemid and 0.5 µg/ml Ethidium Bromide for 3 hours at 37°C and then fixed and stained with Giemsa. ImageJ was used for chromosome counting (Applied StemCell, Inc., CA, USA).

**DNA methylation assays**

Genomic DNA was isolated using the Blood and Cell Culture DNA Mini Kit (Qiagen, CA, USA). Samples were sent to EpigenDx (MA, USA) for determination of % methylation for Oct4, Nanog, and Flk1 promoters.

***In vitro* cardiac differentiation of MiPS**

MiPS (2x10^6^ cells/10 ml medium) were grown in suspension for 3 days in high glucose DMEM containing 10% FBS, 0.5% penicillin/streptomycin, 0.1 mmol/L non-essential amino acids, and 0.1 mmol/L mercaptoethanol)^10^. After 3 days in suspension, embryoid bodies (EBs) were plated on 0.1% gelatin-coated tissue culture dishes (500-1000 EBs/10 cm dish) and cultured for another 7-10 days. Spontaneously contracting regions were mechanically isolated and dissociated for further experiments.

**Transmission electron microscopy**

Conventional transmission electron microscopy was performed on the myocardial tissue samples which were cut into 1-mm^3^ cubes and fixed in 3% glutaraldehyde in 0.1M cacodylate buffer (pH 7.4) at 4°C for 24-hours as described earlier^10^. The tissue samples were then post-fixed in 1% OsO4 followed by uranyl acetate staining for 30-minutes at room temperature. Dehydration of the tissue samples was carried out in a serial ethanol concentrations of 50%, 70%, 90%, and 100% and embedded in Epon-812. Thin sections (~60nm) were cut with an ultra-microtome and after staining with lead acetate, viewed in a JEOL transmission electron microscope.

**miR expression profiling**

Total RNA was extracted from samples using the *miR*Vana^TM^ miR Isolation Kit (Ambion, TX, USA). MicroRNA microarray expression profiling services were provided by Exiqon.

***In vitro* studies of anoxia-induced angiogenic factors**

Native MSC, MiPS, MiPS-CP, MEF and FiPS were serum-starved for overnight. Cells were treated with two 30-minute cycles of anoxia with one 10-minute re-oxygenation in between. Control cells for each group were maintained at normal oxygen levels. The cells were immediately harvested and analyzed by RT-PCR for fibroblast growth factor (FGF) and vascular endothelial growth factor (VEGF) expression.

**Experimental animal model of coronary artery ligation and MiPS transplantation**

An acute myocardial infarction model was developed in 8-12-week-old immunocompetent female C57BL/6 mice^8^. Animals were anesthetized (Ketamine/Xylazine 0.05 ml intraperitoneally) and intubated. The coronary artery was permanently ligated and the ischemic myocardium was confirmed by color change in the left ventricular wall. Ten minutes following coronary artery ligation, cells were injected into the left ventricle under direct vision. Animals were grouped to receive intramyocardial injections of 20 µl basal DMEM without cells (control), or 2x10^5^ MSC (group-1), MiPS (group-2), and MiPS-CP (group-3), FiPS (group-4), or MEF (group-5). The cells were labeled with red fluorescent Q-tracker®-625 (Invitrogen, CA, USA) to study the fate of the cells post transplantation in the infarcted heart.

**Histochemical and immunohistochemical studies**

Histological and immunohistological studies were performed as previously described^10^. To measure infarction size, hearts were arrested in diastole by intravenously injecting them with cadmium chloride followed by fixation in 10% formalin. The hearts were cut transversely and embedded in paraffin. Histological sections were cut at 6 µm thickness and were stained with Masson’s trichrome staining to visualize fibrosis. Cryosections were fixed with 4% paraformaldehyde in PBS for 10 minutes. After fixation, slides were incubated with CAS-BLOCK (Invitrogen, CA, USA) for 1 hour at room temperature. Slides were then incubated with primary antibody overnight at 4˚ C (Supplementary Table-II). Slides were then washed in PBS and the primary antigen-antibody reaction was detected using goat anti-rabbit Alexa Fluor-488 conjugated secondary antibody and goat anti-mouse Alexa Fluor-488 conjugated secondary antibody (1:200, Invitrogen, CA, USA).

**Transthoracic echocardiography**

The animals were anesthetized, the chest was shaven and after application of Acoustic gel, transthoracic echocardiography was performed using HDI-5000 SONOS-CT (HP) ultrasound machine with a 7-MHz transducer as described earlier^10^. The heart was imaged in the two-dimensional mode in the parasternal long-axis and/or parasternal short-axis views which were subsequently used to position the M-mode cursor perpendicular to the ventricular septum and LV posterior wall. Measurements were obtained from 4-5 consecutive heart cycles. Measurements of ventricular septal thickness (VST), LV internal dimension (LVID), and LV posterior wall thickness (LVPW) were obtained from two-dimensional M-mode images during systole and diastole. The average value from all measurements were used to calculate the indices of LV contractile function including LV fractional shortening (LVFS) and LV ejection fraction (LVEF) as: LVFS= (LVEDD-LVESD)/LVEDd x100 and LVEF=[(LVEDD^3^-LVESD^3^)/LVEDD^3^]x100 and expressed as percentages.

**Legends to the Supplementary Figures**

**Supplementary Figure-I. Characterization of MSCs that were used to generate iPS cells.** Native MSCs from the bone marrow of Oct-4 GFP mice were analyzed for MSC-specific surface markers by flow cytometry. The cells were 86.6% and 88.3% pure for CD29 and CD44, respectively. The MSCs also had very low expression of hematopoietic markers CD31 (1.1%) and CD45 (1.0%).

**Supplementary Figure-II. Exogenous transgene expression was abrogated after reprogramming of MSCs.** (A) Five days after the initial transduction with Oct4, Sox2, Klf4, and cMyc (d5 OSKM), transgene expression was still high with low levels of endogenous factor expression. In the fully reprogrammed MiPS clones endogenous expression of the four stemness factors was upregulated while exogenous transgene expression was diminished. Densitometry was done for gene expression in MiPS relative to day 5 of initial transduction. GAPDH was used as a loading control. (B) Percent methylation for Oct4, Nanog, and Flk1 promoters. Methylation of Oct4 and Nanog, respectively, in native MSC (63.9±9.1%; 43.9±15.9%) and MEF (58.6±6.3%; 53.2±11.0%) was significantly decreased in MiPS (4.1±0.4%; 10.5±2.7%) and FiPS (5.0±0.9%; 6.9±0.8%), similar to ESC (2.5±0.2%; 6.2±2.0%). Methylation of Flk1 was similar in both MiPS (1.9±0.6%) and FiPS (2.2±0.6%), but was significantly reduced in MiPS compared to native MSC (5.8±1.0%) and no significant changes were observed in FiPS *vs.* MEF (3.9±0.6%). (C) Fluorescence immunostaining of MiPS confirming endogenous expression of pluripotency markers Oct4 and Sox2 (red). Nuclei were visualized by DAPI staining (blue) and the two images were merged (original magnification=20x1.6).

**Supplementary Figure-III. DNA methylation activity and karyotyping analysis of MiPS.** (A) Analysis of DNA methyltransferase activity (DNMT) confirmed that in MiPS, DNMT activity was significantly decreased (n=3; **p*=0.001 *vs* MSC) similar to activity levels that are observed in ESCs (#*p*=0.003 *vs* MSC). There was no significant difference in DNMT activity between ESC and MiPS (*p*>0.05). Upon differentiation of MiPS into cardiac progenitors, DNMT activity was significantly increased to promote epigenetic silencing of pluripotency genes (†*p*=0.001 MiPS *vs* MiPS-CP; ‡*p*=0.002 ESC *vs* MiPS-CP). (B) Karyotype analysis confirmed that MiPS displayed a normal number of chromosomes.

**Supplementary Figure-IV. Teratoma formation assays in immunodeficient mice . (A)** Tumors were observed in immunodeficient mice 2 weeks after injection with MiPS (n=3; 500,000 cells per animal). (B) Histological sections of the teratomas revealed derivatives of the three germ layers were present.

**Supplementary Figure-V. Teratogenicity of MiPS transplanted into immunocompetent mice.** (A) Cardiac tumors were observed in mice transplanted with undifferentiated MiPS after 4 weeks. Oct4 was not downregulated after transplantation in some cells as they were still Oct4-GFP+ (green). (B) Immunohistological sections of the cardiac tumors confirmed the presence of markers specific to all three germ layers. Transplanted cells that were labeled with Qdots (red) were co-localized with markers for desmin (green), alpha fetoprotein (AFP; green), and beta-tubulin (green) thus confirming that the MiPS contributed to tumor formation. Nuclei were stained with DAPI (blue) for visualization (original magnifications: A=20x; B=100x).

**Supplementary Video 1: MiPS-derived EBs formed spontaneously beating clusters.** We observed spontaneously beating regions of cells after 10 days of differentiation of MiPS.
